# The Use of D-Optimal Mixture Design in Optimising Okara Soap Formulation for Stratum Corneum Application

**DOI:** 10.1155/2014/173979

**Published:** 2014-12-08

**Authors:** Farrah Payyadhah Borhan, Siti Salwa Abd Gani, Rosnah Shamsuddin

**Affiliations:** ^1^Halal Product Research Institute, Universiti Putra Malaysia (UPM), Putra Infoport, 43400 Serdang, Selangor, Malaysia; ^2^Centre of Foundation Studies for Agricultural Science, Universiti Putra Malaysia (UPM), 43400 Serdang, Selangor, Malaysia; ^3^Department of Chemistry, Faculty of Science, Universiti Putra Malaysia (UPM), 43400 Serdang, Selangor, Malaysia; ^4^Department of Process and Food Engineering, Faculty of Engineering, Universiti Putra Malaysia (UPM), 43400 Serdang, Selangor, Malaysia

## Abstract

Okara, soybean waste from tofu and soymilk production, was utilised as a natural antioxidant in soap formulation for stratum corneum application. D-optimal mixture design was employed to investigate the influence of the main compositions of okara soap containing different fatty acid and oils (virgin coconut oil A (24–28% w/w), olive oil B (15–20% w/w), palm oil C (6–10% w/w), castor oil D (15–20% w/w), cocoa butter E (6–10% w/w), and okara F (2–7% w/w)) by saponification process on the response hardness of the soap. The experimental data were utilized to carry out analysis of variance (ANOVA) and to develop a polynomial regression model for okara soap hardness in terms of the six design factors considered in this study. Results revealed that the best mixture was the formulation that included 26.537% A, 19.999% B, 9.998% C, 16.241% D, 7.633% E, and 7.000% F. The results proved that the difference in the level of fatty acid and oils in the formulation significantly affects the hardness of soap. Depending on the desirable level of those six variables, creation of okara based soap with desirable properties better than those of commercial ones is possible.

## 1. Introduction

Cosmetics are daily applied on a part or all parts of human body; thus, they require special knowledge and care in designing formulations. Cosmetics are classified into few categories such as hair care products, skin care products, makeup products, perfumes, and colognes. Currently, the industry of cosmeceuticals is tremendously increasing. The availability of new functional ingredients from botanical or chemical sources, increasing in consumer demands, and better understanding of skin physiology create eagerness among the formulation scientists to find and develop the best solution for various skin problems. However, consumers are now looking for low-cost natural based cosmetic products.

With increasing demands and more soybean-based product industries leaving large quantities of soybean waste annually in Asian countries such as Malaysia, Japan, Korea, and Hong Kong, it is leading to environmental pollution caused by improper disposal problems. Residues and waste from soymilk and tofu production are known as okara. Okara can be classified as natural antioxidants, as there is a high extraction of total phenolic contents and there are high radical scavenging activities in okara [1]. The various components in soy extract exhibit anticarcinogenic activity [2]. In addition, soybean extracts contain various phytochemicals that refer to naturally occurring chemical substances in plants that have biological effects on skin, such as isoflavones and other phenolic compounds, saponins, phytic acids, and phytosterol [3, 4]. Studies have reported that soy isoflavones and soy protease inhibitor are in the top five common cosmeceutical active ingredients based on their antioxidant effects and ability to prevent the signs of skin aging [5].

There are also studies that prove the effectiveness of soybean phytosterol in recovering the stratum corneum (SC), the thin uppermost layer of skin, when subjected to chemical injury [6]. Thus, it is suitable to act as a substitute for active ingredients in cosmeceutical products. However, the available information on this aspect is still limited. In fact, there is no published data on the utilisation of okara in a cosmeceutical realm. Therefore, considering the high content of the beneficial compounds in okara and the environmental effect if they are not managed properly, the development of cosmeceutical products such as soaps may aid in reducing this problem.

Soap, chemically known as alkyl carboxylate, is probably the oldest skin cleanser to be used for cleaning purposes, as cleansing is crucial for hygiene and health [7]. In past decades, soap for skin cleanser technologies has evolved from merely cleansing to providing moisturizing, mildness, and other multifunctional effects on the skin, especially at the SC [8], which is easily exposed to the adverse environment that leads to clogged pores and dead layers of cells if they are not completely removed. In addition, soap formulation has undergone alteration and enhancement by the addition of active ingredients that act as antioxidants, such as ascorbic acid, palmitate, tocopherol acetate, niacinamide, and other natural antioxidants from plant extracts, which promote healthy skin [5]. For a daily personal cleanser such as soap, it is desirable to have those benefits that will further provide homogenous cleansing and moisturizing without irritation.

The development of cosmeceutical products is an intricate task, as optimizing the cosmeceutical formulations involves many “ingredients” and “constituents”; meanwhile, it must be desirable [9]. In formulating a soap bar, some sort of system containing cleansing surfactant, moisturizers, chelating agent, whitening agent, antioxidant, lather enhancer, fragrance, and dye is needed in achieving targeted efficacies and effects on the skin. In formulating a soap bar with natural antioxidants for SC application, products must have equivalent hardness and softness in order to be economically attractive and physically stable, so as to avoid product damage and changing in appearance during storage, owing to melting or high temperatures. Moreover, the hardness of soap is important, not only from the customer's point of view, but also from a processing point of view. In addition, as hard soap will have a lower solubility, it will prolong the shelf life of the soap itself and vice versa for softer soap, but it will also make the soap easy to crack [10].

Multivariate statistical techniques, such as D-optimal mixture design, were used to obtain the desired characteristics and functional stability [11–14]. There are many types of statistical techniques used to optimise analytical procedures, such as response surface methodology and Box-Behnken, but D-optimal mixture design is commonly and widely used in product formulation, especially in the food [15, 16] and pharmaceutical and cosmeceutical industries. The main advantage of using D-optimal mixture design is reported to be the reduction in the number of experimental runs needed to evaluate multiple variables [13, 17]. Moreover, it has the ability to identify interactions statistically, which is able to overcome the shortcomings of the traditional formulation method.

In this present study, okara was utilised as a natural antioxidant to develop soap formulations for SC applications. D-optimal mixture design has been proposed for evaluating the influence of individual factors, such as the different compositions of oil or fatty acids and their interactive effects, as well as obtaining an optimal formulation with desirable physical characteristics, especially in terms of the hardness of the soap.

## 2. Materials and Methods

### 2.1. Materials

Samples of olive oil were purchased from Coreysa, Spain. Samples of palm oil were purchased from Yee Lee Corporation, Berhad, Malaysia. Samples of castor oil and virgin coconut oil were purchased from Euro Chemo-Pharma Sdn Bhd, Malaysia. Cocoa butter was obtained from Making Cosmetics Inc., USA. Sodium hydroxide (NaOH) was obtained from R&M Chemical (Essex, UK). Water was deionised by a Purelab Flex (Elga, Albania) filtration system. All other chemicals were of analytical and cosmetic grades.

### 2.2. Preparation of Plant Materials

Soybean was obtained from a local soybean-milk manufacturer in Serdang, Selangor, Malaysia, and was cleaned and soaked in distilled water for 7-8 hours. The wet soybean samples were then ground by a food processer (Khind, Malaysia). The soymilk was extracted by hand pressing and filtered by cheesecloth. The soybean residue was taken and dried in a Scanvac Coolsafe freeze dryer (Labogene, United Kingdom) at −110°C for six days and then stored and kept in airtight container at room temperature to avoid compound degradation and for further analysis.

### 2.3. Preparation of the Soap Formulation Containing Soybean Waste

Soap samples were prepared according to ingredients formulated from the design matrix. The soybean-waste soap was formulated using a mixture of oils, fatty acids, and natural antioxidants, with additional ingredients by a saponification process. The method in Mensah and Firempong in 2011 [18] was modified and used for okara soap preparation. The fats and oils were weighed out and transferred into a 500 mL beaker and heated at 82°C with continuous stirring, using an overhead stirrer (IKA RW 20 Digital, Nara, Japan). The temperature of the soap mixture was not allowed to exceed 82°C or to fall below 71°C. The saponification reaction was initiated by adding half of the NaOH and, after 5 min, the other half was added along with EDTA. The mixture was stirred until it turned into a pudding-like consistency, which indicated completion of the saponification process. The temperature of the mixture cooled to 65–60°C, where other necessary ingredients were added, and the temperature was then further cooled to 40°C, where the soybean waste was added to the mixture. Then, 50 g of the soap paste was moulded using a wooden mould and allowed to cool in a refrigerator (to 4°C) overnight before demoulding. The finished moulded soap samples were each cut to dimensions of 7.5 cm breadth, 4.0 cm length, and 10 mm height. The finished soap samples were air-dried on plastic trays and conditioned at ambient temperature (25 ± 2°C) for 3 weeks before they were analysed.

### 2.4. Experimental Design

A six-factor D-optimal design mixture was employed to determine the effect of the blend of oil (A–D), fatty acid (E), and natural antioxidant (okara, F) on response variables, including hardness (*Y*) of the soap formulation. The coded independent variables for mixture design are listed in Table 1. The design matrix with a total of 19 runs was generated using Design-Expert 7.0.0 software (Stat. Ease Inc., Minneapolis, USA), as shown in Table 2. Each design was evaluated separately, based on the influence of each composition of variables towards the response (*Y*). The composition of each run was carried out in a randomised order, according to the D-optimal model design in order to minimise the effect of unexplained variability on the actual response, owing to the extraneous factor.

### 2.5. Determination of the Hardness of the Okara Soap

The hardness of the soybean-waste soap was measured using a TA HDplus texture analyzer (Stable Micro System Ltd., Surrey, UK) with a cell load of 500 N and using needle geometry. The probe used was a stainless P/2:2 diameter needle cylinder. Hardness was reported as the maximum penetrating force (N) required for the needle to penetrate through a sample (70.5 mm × 40 mm, depth 10 mm) at 25°C, over a distance of 8 mm at a constant speed of 10 mm/s.

### 2.6. Statistical Analysis

The optimum conditions for the causal factor variables were ascertained by conducting D-optimal mixture design in order to predict the effects of the variation of ingredient compositions towards the response penetration force, which indicates the hardness of the soap (*Y*_1_). The statistical parameters, including the adjusted multiple correlation coefficients (adjusted *R* ^2^), multiple correlation coefficient (*R* ^2^), coefficient of variation (C.V.), lack of fit, regression (*P* value), and regression (*F* value), were used to evaluate and select the best-fitting mathematical method [19–21]. The design was expressed by the polynomial regression equation to generate the model as shown as follows:

(1) Yi=β0+β1x1+β2x2+β3x3+β4x4−β11x12 +β22x22+β33x32+β44x42+β12x1x2 +β13x1x3+β14x1x4+β23x2x3+β24x2x4+β34x3x4,

where *Y*_*i*_ is the predicted response and *β*_0_, *β*_*i*_, and *β*_*ii*_ are the linear coefficient, quadratic coefficient, and interaction coefficient, respectively. The suitable polynomial equations for the design, such as linear, quadratic, or special cubic models, were chosen according to the fittest model.

For a better understanding and to view the interaction of the response variables and causal factor variables, the mixture design space and three-dimensional (3D) contour plots of the fitted polynomial regression were generated. Graphical and numerical optimizations were conducted to obtain optimum conditions and to predict values for the desirable response using a response optimizer.

### 2.7. Verification of the Model

A few random formulations were prepared to validate the models. Verification of the model was carried out by comparing the experimental values with the predicted value obtained from the final response regression equations [22]. This step is of utmost importance to check the adequacy of the final reduced models. The recommended optimum composition was also performed to verify the optimum values predicted by the model.

## 3. Result and Discussion

### 3.1. Screening of the Variables

**Figure 1:**
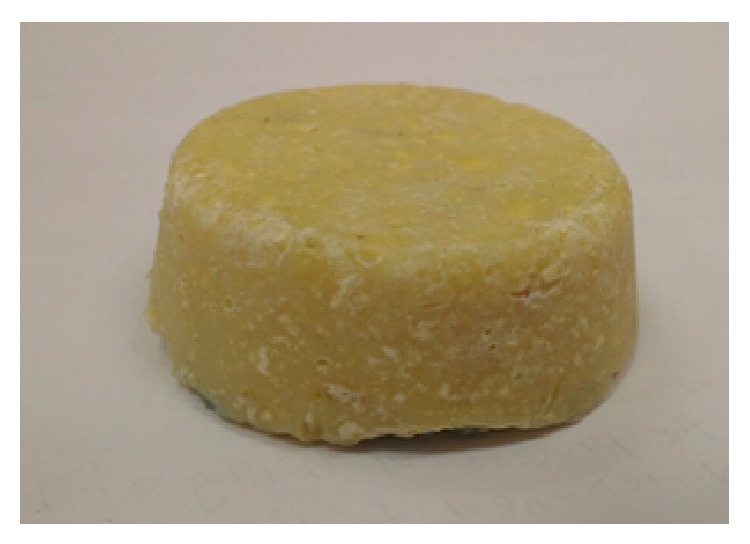
Okara soap.

A preliminary study was carried out to evaluate the levels of independent variables. Based on the resultant data of the construction and analysis of mixture designs upper- and lower-bound restrictions on the component proportion were determined. Soap formulations showed hardness in the range of 200 N–600 N in the range of virgin coconut oil A (24–28% w/w), olive oil B (15–20% w/w), palm oil C (6–10% w/w), castor oil D (15–20% w/w), cocoa butter E (6–10% w/w), and okara F (2–7% w/w). Table 1 presents the coded levels of the variable used in the formulation. The construction and analysis of mixture designs require characterisation by upper- and lower-bound restrictions on the component proportion. Table 2 presents the mixture design for the 19 formulations, which underwent analysis.  Figure 1 depicted the okara soap after 3 weeks of drying.

### 3.2. Fitting the Response Surface Model

Table 3 presents the experimental data of all model formulations obtained for the response *Y*_1_ based on the D-optimal mixture design matrix. The variation in penetration force (N) indicates the hardness of the soap, the values of which were predicted by utilising D-optimal mixture design, as the response (*Y*_1_) is an important characteristic of the soap. The obtained experimental data were statistically analysed. Statistical analysis (analysis of variance; ANOVA) was utilised to determine the best-fitted model for the six independent variables. The analysed statistical parameter values, including the adjusted multiple correlation coefficient (adjusted *R* ^2^), multiple correlation coefficient (*R* ^2^), coefficient of variation (C.V.), and lack of fit, generated by Design-Expert software were used to evaluate and select the best-fitting mathematical method, which are shown in Table 4.

In order to evaluate the contribution of each of the six components and the quantitative effects of the different proportions of the formulation variables on the response penetration force (*Y*), the response surface models were calculated with Design-Expert software. The final equation for the model describing the penetration force of the soap can be written as

(2) HardnessY/N=+544.2X1A−433.3X2B +501.0X3C+577.6X4D +498.4X5E+505.3X6F.

The statistical value analysed by ANOVA, showing the assumed mathematical model, was significant and valid for the response (*Y*_1_). In this work, the response mixture analysis demonstrated that the linear polynomial regression, utilised for the hardness of soap (N), has high coefficient of determination (*R* ^2^ = 0.9578). The obtained coefficient of the determination value of the response indicates rightness of the model and showed that more than 90% of the variation of the hardness could be described by mixture models, which are a desirable physical characteristic of the prepared soap formulation. In addition, it was observed that the lack of fit had no indication of significance (*P* < 0.05) for the final reduced model [17, 23, 24], therefore, indicates the fittest model towards the response.

A positive term in the linear regression equation represents an effect that favors optimisation, owing to synergistic effects, whereas a negative term reveals an antagonistic effect between the factors and the response [24, 25]. From the final mathematical equation of the hardness, it showed that virgin coconut oil (A) and castor oil (D) show the greatest influence on the response.

**Figure 2:**
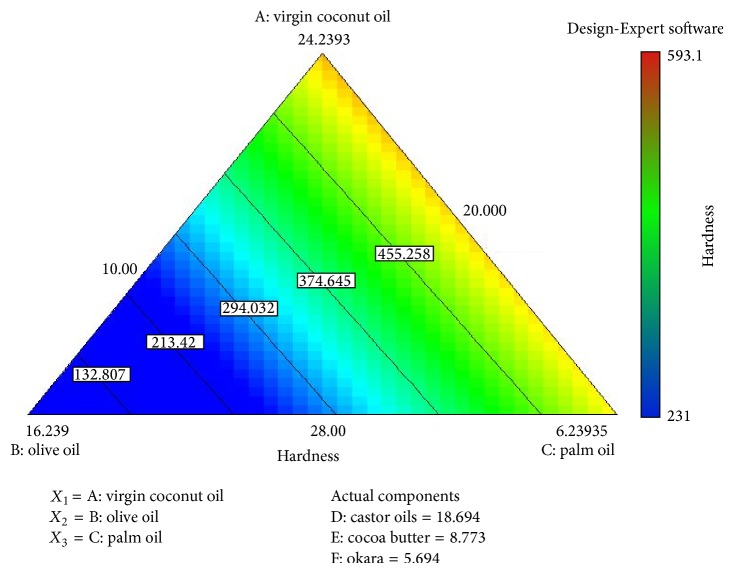
Contour plot: the effect of the different fatty acid and oils on the response, hardness (N).

**Figure 3:**
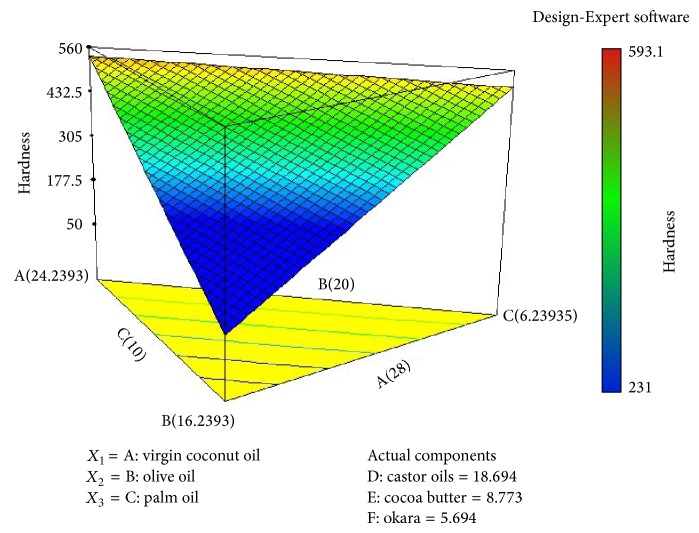
Three-dimensional diagram illustrating the effect of the different fatty acid and oils on the response (*Y*), hardness (N).

As shown in Figure 2, the higher the percentage of olive oil in the soap formulation, the more force needed to penetrate the soap at certain constant distances. There are few possible explanations behind this observed result. Olive oil is categorised as both a soft and hard oil [26]; the uses of soft oil in soap formulation will produce good lathering ability, improved solubility of soap in cold water [27] and have a moisturizing effect on the skin [26]. Olive oil also has a higher percentage of unsaturated fatty acids. Generally, soaps made from a high percentage of these oils will produce a softer soap, but the only exception to this rule is olive oil. Soaps made from a high percentage of olive oil are soft upon unmoulding, but will be really hard after completely dry [26]. The 3D contour plot in Figure 3 reveals the effects of a high percentage of olive oil on the response, that is, the higher force needed to penetrate the soap. To overcome the problem with a hard soap bar, the soap formulation needs to be blended with more soft oil, such as castor oil.

As shown in Figure 3, the hardness of soap increases with the increase of virgin coconut oil. The increase in the penetration force indicates that the soap is harder, as more force is utilised to penetrate certain distances. There are few possible reasons for these observed results. According to the studies by Marina et al. [28] in 2009, the dominant fatty acid in coconut oil was lauric acid (C 12 : 0), the saturated fatty acid. Generally, oils with higher saturated fatty acids will produce hard soaps [10]. However, virgin coconut oil not only affects the hardness of the soap, but also added some therapeutic values, especially for skin, such as antioxidant, antibacterial, and antiviral activities [20].

Castor oil is considered a soft oil that will produce softer soaps. The major fatty acid in castor oil is unsaturated fatty acid ricinoleic acid [21]. In soap making, a higher composition of this type of oil will provide a conditioning effect to the skin and it will be more bubbly and form a creamy lather. This oil component also acts as a stabilising system to the formulation, as it will balance the hardness of the soap. This explanation supported the mathematical equation and the 3D contour plot, where castor oil had the greatest potential on the response, where the higher the percentage of this oil, the lower the penetration force, which indicates the soap is too soft and it need to be stabilised with the hard oil,virgin coconut oil.

**Figure 4:**
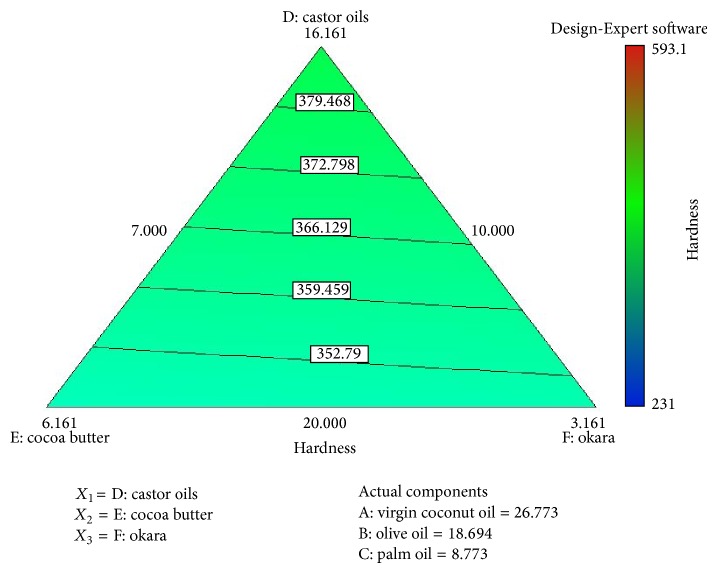
Contour plot that illustrates the relationship between component palm oil, cocoa butter, and okara towards the response, hardness (N).

**Figure 5:**
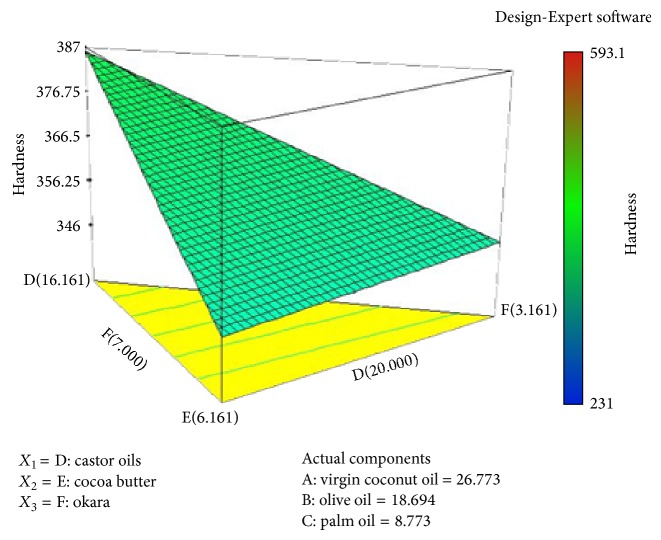
Three-dimensional contour plot illustrating the interaction of palm oil, cocoa butter, and okara towards the response (*Y*), hardness (N).

Figure 4 shows the interaction between okara, palm oil, and cocoa butter. There are minimum effects between these components and the response. The increased percentage of each of these components gives equal effects to the response. This observation was supported by mathematical equation (2), and there are a few possible explanations for this observation. Soybean waste, or okara, basically acts as a natural antioxidant in this formulation. It also acts as an exfoliator to remove the dead layer of skin, stratum corneum. It also contributes to the hardness of the soap, but with minimal effect, as do cocoa butter and palm oil, because they are all formulated in a small range. The major fatty acid in cocoa butter is oleic acid, followed by stearic acid and palmitic acid, which are also saturated fatty acids. Cocoa butter helps to produce a harder bar of soap that has a rich, creamy lather and has more of an aftereffect on the skin, such as a moisturizing effect. This is proven in the 3D plot in Figure 5, where the increase or decrease of the composition implies a minimum effect towards the response.

In the preliminary screening experiment (data not shown) of five different commercial soaps, the range of hardness was shown to be in the range 1000–2000 N. This shows that most of the commercial soaps are harder bars. There are a few possible reasons for this observation, because all of the tested commercial soaps were using only sodium palmate and sodium palm kernelate as the main ingredients. The harder the bar, especially those made mainly of palm- and palm-kernel-based oils, the higher the tendency to experience supercracking effects [10, 29]. In addition, a higher composition of palm-based oils will increase the soap erosion rate and dry the skin [30]. In this work, we try to obtain the optimum hardness in the range 550–650 N by adjusting the composition of oils and fatty acid.

### 3.3. Validation for Verification

The predicted and actual values of the responses were compared in order to check the adequacy of the surface response equation. The optimised formulation of okara soap had a hardness value of 593.1 N. As shown in Table 5, no significant (*P* > 0.05) difference was noted and the results were found to be in good agreement with the predicted values.

## 4. Conclusions

The present study showed D-optimal mixture design is an effective and beneficial tool for carrying out the optimization study of okara soap formulation for stratum corneum application by combining independent variables: virgin coconut oil, olive oil, cocoa butter, palm oil, okara, and castor oil. The influences of different variables, including different compositions of oil and fats on the hardness of soap, were investigated. A linear mathematical model was suggested for the response, hardness (N). Analysis of variance corroborates the accuracy of the model, using a high *F* value (59.50) and a very low *P* value (<0.0001), a nonsignificant lack of fit, and a coefficient of determination of *R* ^2^ = 0.9578. It was concluded that D-optimal mixture design with multiple-response optimisation utilising a polynomial equation can be successfully used in optimising the okara soap formulation with desirable hardness. This research provides a guideline on improving specific desirable characteristics by using D-optimal design which is capable of studying many variables simultaneously. The research finding also provides a guideline on the effects of ingredients towards the physical properties especially in cosmeceutical products.

## Figures and Tables

**Table 1:** Summary of variables for the mixture design.

	Causal factor variables^1,2^	Coded level of variables (%)	
		Low	High
A	Virgin coconut oil	24.00	28.00
B	Olive oil	15.00	20.00
C	Palm oil	6.00	10.00
D	Castor oil	15.00	20.00
E	Cocoa butter	6.00	10.00
F	Okara	2.00	7.00

(1) Total amount of soybean-waste soap formulation was 50 g, composed of virgin coconut oil (A), extra virgin olive oil (B), palm oil (C), castor oil (D), cocoa butter (E), okara (F), and 12.6% of other necessary ingredients, incorporated in the appropriate amounts to make 100% for each formulation.

(2) A + B + C+ D + E + F = 87.4% + 12.6% (other ingredients) = 100%.

**Table 2:** Matrix of D-optimal mixture design.

Experiment number	Block	A	B	C	D	E	F
1	Block 1	28.00	20.00	9.99	19.16	6.00	4.25
2	Block 1	27.98	20.00	6.00	20.00	8.29	5.13
3	Block 1	28.00	20.00	9.99	19.16	6.00	4.25
4	Block 1	27.18	19.45	9.02	19.78	9.97	2.00
5	Block 1	27.18	19.45	9.02	19.78	9.97	2.00
6	Block 1	24.02	20.00	9.90	16.49	10.00	7.00
7	Block 1	28.00	20.00	6.06	18.49	7.85	7.00
8	Block 1	26.54	19.99	10.00	16.24	7.63	7.00
9	Block 1	28.00	20.00	10.00	15.65	10.00	3.76
10	Block 1	28.00	20.00	8.29	16.77	8.39	5.95
11	Block 1	25.07	20.00	10.00	18.17	8.88	5.28
12	Block 1	28.00	20.00	6.43	15.98	10.00	7.00
13	Block 1	28.00	19.79	7.21	18.07	10.00	4.33
14	Block 1	24.82	18.87	6.71	20.00	10.00	7.00
15	Block 1	24.02	20.00	9.90	16.49	10.00	7.00
16	Block 1	27.17	17.91	10.00	17.89	9.06	5.37
17	Block 1	27.03	18.54	8.19	20.00	9.07	4.58
18	Block 1	28.00	17.69	6.54	18.96	10.00	6.21
19	Block 1	26.35	18.45	10.00	19.57	6.06	6.96

**Table 3:** Experimental data obtained for the response.

Experiment number	Response (*Y*)
1	506.2
2	507.5
3	504
4	469.4
5	418.1
6	523.6
7	494
8	593.1
9	544.1
10	563.6
11	530.2
12	510.2
13	497.8
14	402.3
15	573.4
16	258.7
17	326.7
18	239.8
19	305.2

**Table 4:** Analysis of variance (ANOVA) for the model.

Response variable	PRESS	C.V. (%)	*F* value	*P* value	*R* ^2^	Adjusted *R* ^2^	Pred. *R* ^2^	Standard deviation
*Y*_1_	184.19	5.58	59.05	<0.0001	0.9578	0.94	0.91	2.58

**Table 5:** Predicted and observed values for the optimised formulation.

Response	Predicted	Observed
*Y*: hardness (N)	542.53	541.62
